# The Fungal and Protist Community as Affected by Tillage, Crop Residue Burning and N Fertilizer Application

**DOI:** 10.1007/s00284-025-04112-5

**Published:** 2025-02-19

**Authors:** Luc Dendooven, Valentín Pérez-Hernández, Selene Gómez-Acata, Nele Verhulst, Bram Govaerts, Marco L. Luna-Guido, Yendi E. Navarro-Noya

**Affiliations:** 1 Laboratory of Soil Ecology, Department of Biotechnology and Bioengineering, Cinvestav, Mexico City, Mexico; 2 Laboratorio de Interacciones Bióticas, Centro de Investigación en Ciencias Biológicas, Universidad Autónoma de Tlaxcala, Tlaxcala, Mexico; 3 International Maize and Wheat Improvement Center (CIMMYT), El Batán, Texcoco, Mexico; 4 Cornell University, Ithaca, USA

## Abstract

The bacterial community in soil is often affected by agricultural practices, but how they affect protists and fungi is less documented. Soil from treatments that combined different N fertilizer application rates, tillage and crop residue management was sampled from a field trial started by the International Maize and Wheat Improvement Center (CIMMYT) at the ‘Campo Experimental Norman E. Borlaug’ (CENEB) in the Yaqui Valley in the northwest of Mexico in the early 1990s, and the fungal and protist community determined. Tillage, residue burning, and N fertilizer application had no significant effect on the fungal and protists alpha diversity expressed as Hill numbers and no significant effect on the fungal and protist community structure considering all species. The relative abundance of plant pathogens and undefined saprotrophs as determined with FUNGuildR increased significantly with tillage, while that of dung-plant and dung-soil saprotroph, and plant pathogens by burning (*P* < 0.05). It was found that the protists and fungal community structures were not altered by different agricultural practices, but some fungal guilds were, i.e., plant pathogens and saprotrophs, which might affect soil organic matter decomposition, nutrient cycling and crop growth.

**Supplementary Information:**

The online version contains supplementary material available at 10.1007/s00284-025-04112-5.

## Introduction

Microbial communities are affected by different factors, such as soil characteristics, climate and vegetation, and also by agricultural practices [1]. Each of these factors *in se* and interactions between them define the microbial communities. This makes it often difficult to determine how each of these factors affect the microbial community structure, but the effect of agricultural practices on the microbial community can be studied if the climatic conditions and crops are similar in the studied agroecosystem. As such, long-term field experiments are ideal to study the effect of different agricultural practices, e.g., fertilizer application, tillage, residue burning, on soil microbial communities [2].

The International Maize and Wheat Improvement Center (CIMMYT) started different field experiments in Mexico to study factors that affect crop production. One of these long-term field experiments started in 1992 is situated at the experimental station ‘Campo Experimental Norman E. Borlaug’ (CENEB) in the north coastal province of Sonora and investigates the effect of tillage practices, burning and N fertilizer application on yields of wheat (*Triticum durum* L.) and maize (*Zea mays* L.) [3]. Grahmann et al. [4] reported that yields of durum wheat increased with increased N application rates, e.g., 2.33 t ha^−1^ when no fertilizer was applied, 6.31 t ha^−1^ when 150 kg N ha^−1^ and 6.95 t ha^−1^ with 300 kg N ha^−1^ in 2010/11 and 2.93 t ha^−1^, 6.77 t ha^−1^ and 7.86 t ha^−1^ in 2011/12. The effect of N fertilizer application rate at the experimental site CENEB, however, was larger than that of tillage-straw management. For instance, Verhulst et al. [5] reported that in permanent beds with straw retained yields were on average 7.31 t ha^−1^, in permanent beds with straw removed 7.24 t ha^−1^ and in permanent beds with straw burned 6.65 t ha^−1^.

In previous research we studied the effect of permanent bed planting and residue management on physical and chemical soil quality in rain fed maize/wheat systems at CENEB which showed that tillage and residue management affected soil characteristics and soil processes [6]. We also found that crop residue management, tillage and fertilizer application, affected the soil bacterial community structure, while the mineralization potential of the soil was preserved [7]. Although we studied how agricultural practices affect factors that might control the bacterial community at CENEB [8], we known less on how crop residue management, tillage and fertilizer application affect microscopic eukaryotes, e.g., protists and fungi, although they play important roles in soil fertility and plant growth. For instance, arbuscular mycorrhizal fungi increase crop yields and shoot biomass as photosynthesis, plant nutrition and stress resistance increases [9]. Fungi are important in the degradation of soil organic material thereby providing nutrients for plants, but some are plant pathogens or antagonistic to pathogens [10]. Protists play important roles as organic matter decomposers, predators and pathogens of plants and animals [11]. Additionally, Guo et al. [12] found that protists might play an important role in plant development as cercozoan protists can increase plant growth, potentially through interactions with plant-beneficial microorganisms through microbiome interactions. There is also a close relationship between the protist community and the fungal community structure. In a study of 156 cereal fields in Europe, Degrune et al. [13] found based on a machine-learning approach that climatic variables and fungal communities are the primary drivers of cercozoan communities, accounting for 70% of their community composition.

How protist and fungi are affected by agricultural practices, such as N fertilizer application, burning or tillage and combinations of them in semi-arid agriculture systems with a wheat–maize crop rotation needs further investigation. Essel et al. [14] studied the effects of tillage and stubble management on the diversity of fungi, chemical property and total carbon emission in the rhizosphere and bulk soils in wheat (*Triticum aestivum* L.) -pea (*Pisum arvense* L.) rotation at pre-harvest in a rainfed long-term tillage-rotation experiment in China established in 2001. They found that the fungal community and the Ascomycota and Basidiomycota phyla did not differ among treatments that included conventional tillage with stubble removed, no-tillage with stubble removed, conventional tillage with stubble incorporated and no-tillage with stubble retained. We postulate that the different agricultural practices will affect the fungal communities in different ways. First, tillage might have a negative effect on the fungal community structure as it breaks up aggregates and disrupts fungal hyphae, but it might help to reduce plant pathogens. Second, burning might alter the fungal community by increasing the soil temperature temporarily and removing crop residue. Third, retaining crop residues will enrich some fungal groups, especially saprotrophs (organic matter decomposers), but might also shelter plant pathogens [15]. To test these hypotheses, soil was sampled from six treatments at CENEB combining different tillage practices (conventional tilled and permanent beds), crop residue management (incorporation, retention and burning) and N fertilizer application (unfertilized and wheat crop fertilized with 300 kg urea-N ha^−1^). Microscopic eukaryotes, fungi and protists (all eukaryotes except plants, fungi and animals were determined by metabarcoding of the 18S rRNA gene. The aim of this study was to determine the effect of i) N fertilizer application (0 kg N ha^−1^ vs 300 kg N ha^−1^), ii) burning (permanent beds with residue left on the soil surface vs permanent beds with residue burned) and iii) tillage practices (permanent beds with all crop residue left on the soil surface vs conventional tilled beds with all crop residue incorporated) on the fungal and protist community structure, and functional guilds [16].

## Materials and Methods

### Long-Term Field Experiment at Ciudad Obregón (CENEB)

The experimental station, Campo Experimental Norman E. Borlaug (CENEB) is located near Ciudad Obregón, Sonora, Mexico (lat. 27.33 °N, long. 109.09 °W, 38 masl). The climate at the station is arid with a monthly average temperature that ranges between 16.1 and 31.1 ºC and a yearly average of 23.7 ºC, while monthly average rainfall varies between 1 and 87 mm with a yearly average of 309 mm (1993–2018). The soil type at the experimental station is a Haplic Vertisol (Calcaric, Chromic) in the world reference base system and a Chromic Haplotorrert in the USDA soil taxonomy system.

Details of the experimental design, agricultural practices, fertilizer application and planting can be found in Chávez-Romero et al. [7] and are schematized in Fig. S1. Briefly, the experimental design consisted of a randomized complete block with a split-plot treatment arrangement and three replications with wheat and maize managed in an annual rotation with wheat planted in the winter and maize in the summer. Six different treatments were sampled and used in this study. They combined different management of soil (conventional tilled beds vs permanent beds) and crop residue (crop residue retained on the soil surface, burned or incorporated), and two application rates of urea as N source, i.e., 0 kg N ha^−1^ vs 300 kg urea-N ha^−1^ y^−1^. The treatments sampled at CENEB and used in this study were: (1) conventional tilled beds, i.e., beds are formed after each crop, all crop residues kept and incorporated by conventional tillage, no N fertilizer application (CBK treatment), (2) the same as CBK but with N fertilizer application at 300 kg urea-N ha^−1^ y^−1^ (CBKN treatment), (3) permanent beds are reshaped as needed, wheat and maize straw burned, no N fertilizer application (PBB treatment), (4) the same as PBB but with N fertilizer application at 300 kg urea-N ha^−1^ y^−1^ (PBBN treatment), (5) permanent beds are reshaped as needed, both wheat and maize straw chopped and left in place, no fertilizer application (PBK treatment), and (6) the same as PBK but with fertilizer application at 300 kg urea-N ha^−1^ y^−1^ (PBKN treatment).

The 0–15 cm layer was sampled 10 times with a 2 cm ϕ soil auger during the wheat crop cycle (April 11, 2012). The samples taken at each plot were pooled so that 18 different soil samples were obtained (*n* = 18, three plots and that from six treatments). The field-based replication of sampling at CENEB was maintained for soil characterization and extraction of DNA to avoid pseudoreplication.

References to the techniques used to characterize the soil can be found in Chávez-Romero et al. [7]. The pH was measured by mixing a soil sample with distilled water in a 1:2.5 ratio and pH was measured with a potentiometer Mettler Toledo® Model S220 (New York, USA). Another soil sample was brought to saturation with distilled water, centrifuged and the electrolytic conductivity (EC) was measured in the supernatant with a conductimeter Mettler Toledo® Model S220 (New York, USA). The soil particle size distribution was determined by the hydrometer method. Total C was determined with a Thermo Scientific™ FlashSmart™ Elemental Analyzer (Waltham, Massachusetts, USA) following the standard protocol given by the manufacturer, while the inorganic C was measured by adding 20 mL 1 M HCl solution to 2 g air-dried soil and trap CO_2_ evolved in 20 mL 1 M NaOH. The organic C was defined as the difference between the total and inorganic C. Total N was measured by the Kjeldahl method using concentrated H_2_SO_4_, K_2_SO_4_ and HgO to digest the sample. The soil characteristics are given in Table S1 as reported by Jiménez-Bueno et al. [17].

### DNA Extraction and PCR Amplification of Small Subunit rRNA and Analysis of Pyrosequencing Data

Metagenomic DNA was extracted separately from each soil sample. The DNA was extracted from 2.5 g soil (10 times from 0.25 g) using the Power Soil DNA Isolation Kit (MO BIO Laboratories, CA, USA), according to the manufacturer’s instructions. Fragments of the small subunit rRNAs were amplified using the nu-SSU-0817 and nu-SSU-1196 primers for microscopic eukaryotes [18]. The reaction mixture comprised 5 pmol of each primer, 200 µM dNTPs (Invitrogen, Waltham, MA), 1 × buffer, 3.5 mM MgCl_2_, 40 µg of bovine serum albumin (Sigma Aldrich, Saint Louis, MO), and 1 U of Dream*Taq* DNA Polymerase (Thermo Scientific, Waltham, MA) in a total volume of 25 µL. Primers contained the Roche 454 FLX adapters Lib-L and 10-pb barcoded. Amplification conditions included a denaturation step at 95 °C for 10 min followed by 30 cycles at 95 °C for 40 s, at 55 °C for 40 s, at 72 °C for 50 s, and a final step at 72 °C for 10 min. The PCR targeting eukaryotic 18S rRNA were all done in 25 μL reaction using Phusion hot start high fidelity DNA polymerase (FINNZYMES). Amplicon libraries were pooled and purified using the QIAquick PCR Purification Kit (QIAGEN, Germany). Sequencing was done by Macrogen Inc. (Seoul, Korea) on a Roche 454 GS-FLX Titanium™ pyrosequencer (Roche, Mannheim, Germany). Poor quality reads were eliminated from the data sets, i.e., quality score < 25, containing homopolymers > 6, length < 400 nt, and containing errors in primers and tags. Noise from the FLX chemistry was eliminated with the script *denoise_wrapper.py* [19]. It is important to mention that the primers for microscopic eukaryotes nu-SSU-0817 and nu-SSU-1196 might be biased towards fungi amplification. The QIIME version 1.9.0 software pipeline was used to analyse the pyrosequencing data [20]. Denoising algorithms of pyrosequencing reads are better corroborated in this software, while QIIME2 is more focused on Illumina sequencing data.

Operational taxonomic units (OTU) were determined at 97% similarity level with UCLUST algorithm [21]. Chimeras were detected and removed from the data using the Chimera Slayer [22]. Taxonomic assignment of the different microscopic eukaryotes, protist and fungal groups was done using the naïve Bayesian rRNA classifier from the Ribosomal Data Project (http://rdp.cme.msu.edu/classifier/classifier.jsp) at a confidence threshold of 80% against the SILVA database version 132 [23].

Sequences belonging to Fungi were analyzed seperately and sequences belonging to the Rhodophyta, Streptophyta, Metazoa, and Fungi were excluded when analyzing Protist species.

### Statistical Analysis

All statistical analyses were done in R v4.4.1 [24]. A non-parametric analysis was used to determine the effect of agricultural practices (tillage, residue management, N fertilizer application) on the soil characteristics with the WRS2 package (v. 1.1–6) (A collection of robust statistical methods based on Wilcox' WRS functions). Alpha diversity of soil microbial communities, i.e., fungi and protists, was determined based on the Hill numbers at different *q* orders (at *q* = 0, 1 and 2) which modulates the importance given to the abundance of the species. The Hill number at *q* = 0 gives the species richness, *q* = 1 is the Shannon entropy and denotes frequently occurring species and *q* = 2 is the inverse Simpson and characterizes dominant species. They were calculated with the HillR package v. 0.5.3. A non-parametric analysis (t1way test of the WRS2 package, v. 1.1–6) was used to determine the effect of treatment, tillage, residue and N fertilizer on the Hill numbers.

Ordination (principal component analysis (PCA)) and multivariate comparison (perMANOVA) tests were done with converted sequence data using the centred log-ratio transformation test returned by the aldex.clr argument in the ALDEx2 package (v. 1.36.0). Centred log-ratio transformation of all sequence data are necessary as all high-throughput data or compositional. A principal component analysis (PCA) was used to visualize the effect of agricultural practices (tillage, residue management, N fertilizer application) on the fungal and protist community structure, and fungal guilds with the FactoMineR package (v. 2.11) (Multivariate exploratory data analysis and data mining). A perMANOVA test was used to determine the effect of the different agricultural practices (tillage, residue management, N fertilizer application) on the fungal and protist community structure with the Vegan package (v. 2.6–8). Permutation multivariate analysis of dispersion (PERMDISP) was computed using the *betadisper* function from the vegan R package (v2.6–8, Community ecology package) to determine dispersion within treatments when the perMANOVA test indicated a significant effect of agricultural practices (tillage, residue management, N fertilizer application) on the fungal and protist community structure, and fugal guilds.

Guilds were assigned to organisms based on taxonomic classification using the FUNGuild database [16] FUNGuildR package (v. 0.2.0.9000, Look up guild information for fungi). The non-parametric Kruskal–Wallis test was used to determine the effect of the agricultural practices (tillage, residue management, N fertilizer application) on the different fungal and protist groups with the WRS2 package (v 1.1–6). The effect size (the difference between the groups divided by maximum dispersion within the treatment) of the agricultural practices (tillage, residue management, N fertilizer application) on the fungal and protist community structure, and fugal guilds was determined with the WRS2 package (v. 1.1–6). The effect size was plotted (volcano plot) versus the expected p value of the Kruskal–Wallis test for each feature.

### Data Accessibility

Raw sequence datasets were submitted to the NCBI Sequences Read Archive under project PRJNA255111 with accession numbers accession SRR24724994, SRR24724993, SRR24725002, SRR24725001, SRR24725000, SRR24724999,SRR24724998, SRR24724997, SRR24724996, SRR24724995, SRR24724992, SRR24724991, SRR24725008, SRR24725007, SRR24725006, SRR24725005, SRR24725004, SRR24725003.

## Results

### Soil Characteristics

The pH in the soil of CENEB varied between 8.6 and 8.9, while the total C between 9.6 g kg^−1^ soil and 13.3 g kg^−1^ soil (Table S1). The total N was significantly higher in the N fertilized (0.57 g kg^−1^) than in the unfertilized soil (0.48 g kg^−1^) (*P* < 0.05) (Table S2). Leaving the crop residue on the permanent beds increased the soil organic C content significantly and decreased EC and pH compared to conventional tilled beds with the crop residue incorporated (*P* < 0.05). Burning the crop residue reduced the soil organic C content significantly compared to the treatments where it was left on the soil surface, but increased pH and EC significantly (*P* < 0.05).

### Microscopic Eukaryotes

**Fig. 1:**
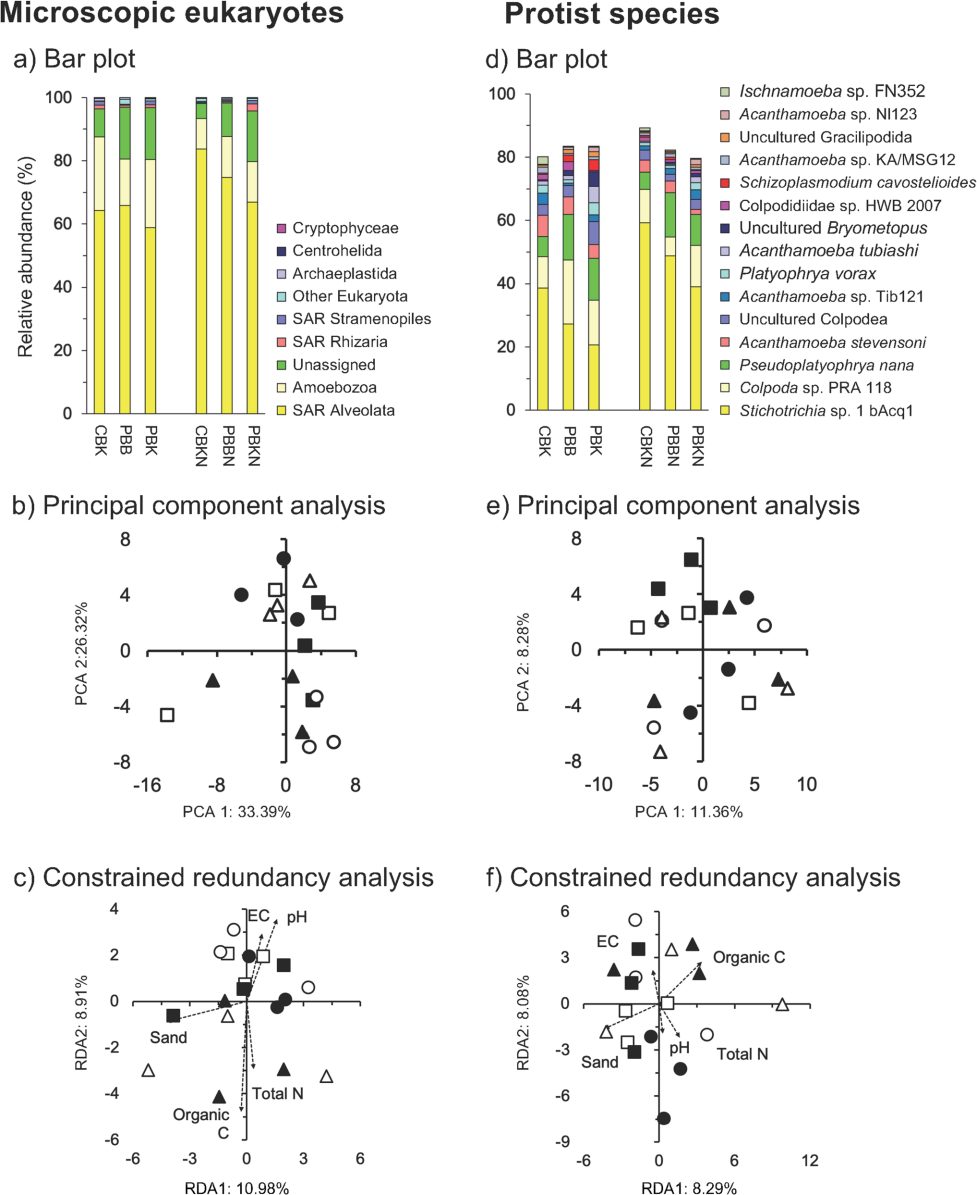
**a** Bar plot, **b** principal component analysis (PCA), **c** constrained redundancy analysis (RDA) with all higher taxonomic levels of microscopic eukaryotes, and **d** bar plot, **e** PCA and **f** constrained RDA with the protist species in soil at the “Campo Experimental Norman E. Borlaug” (CENEB). The treatments were CBK (white square): conventional tilled beds, i.e., beds are formed after each crop, all crop residues kept and incorporated by conventional tillage, no N fertilizer application, CBKN (black square): the same as CBK but with N fertilizer application at 300 kg urea-N ha^−1^ y^−1^, PBB (white circle): permanent beds are reshaped as needed, wheat and maize straw burned, no N fertilizer application, PBBN (black circle): the same as PBB but with N fertilizer application at 300 kg urea-N ha^−1^ y^−1^, PBK (white triangle): permanent beds are reshaped as needed, both wheat and maize straw chopped and left in place, no fertilizer application, PBKN (black triangle): the same as PBK but with fertilizer application at 300 kg urea-N ha^−1^ y.^−1^

Members of Nucletmycea (84.5 ± 10.2%), that include Nuclearia, Fonticula, and Fungi, were the most dominant microscopic eukaryotes with Alveolata (11.1 ± 8.0%) the second most abundant (Fig. 1a). The perMANOVA analysis showed no significant effect of N fertilizer application, burning or tillage on the microscopic eukaryotes and the PCA did not separate the soil communities in the different treatments (Fig. 1b; Table S3). The Mantel test indicated that no soil characteristic was significantly correlated to the microscopic eukaryotes (Table S4). The constrained RDA did not separate the microscopic eukaryotes in the different soils (Fig. 1c). Nitrogen fertilizer application or burning had no significant effect on the microscopic eukaryotes (*P* < 0.05) (Table S5).

### Protists

The Hill number at *q* = 0 of the protist species, i.e., all microscopic eukaryotes except plants, fungi and animals, ranged on average from 24 in the CBKN treatment to 44 in the PBKN treatment, while the number of dominant protist species (Hill number at *q* = 2) from 2 to 8 (Fig. S2a). Treatment, tillage, burning or N fertilizer application had no significant effect on the protists Hill numbers (Table S6).

*Stichotrichia* sp. 1 bAcq1 (Ciliophora, Spirotrichea, 38.97%) was the most abundant protist species (Fig. 1d). The perMANOVA analysis showed no significant effect of N fertilizer application, burning or tillage on the protist species community and the PCA and constrained RDA did not separate the soil communities in the different treatments (Fig. 1e, f; Table S3). The Mantel test indicated that no soil characteristic was significantly correlated to the protist community (Table S4). Agricultural practices had a limited effect on the relative abundance of the protist species (Fig. S3a; Table S5).

### The Fungal Community

The Hill number at *q* = 0 number of the fungal species (ranged from 50 in the CBKN treatment to 70 in the PBKN treatment, while the number of typical species (Hill number at *q* = 1) ranged from 7 in the CBK treatment to 14 in the PBK treatment (Fig. S2b). The number of dominant fungal species (Hill number at *q* = 2) ranged from 4 in the CBK and PBBN treatments to 8 in the PBK treatment. Tillage, burning or N fertilizer application had no significant effect on the fungal Hill numbers (Table S6).

**Fig. 2:**
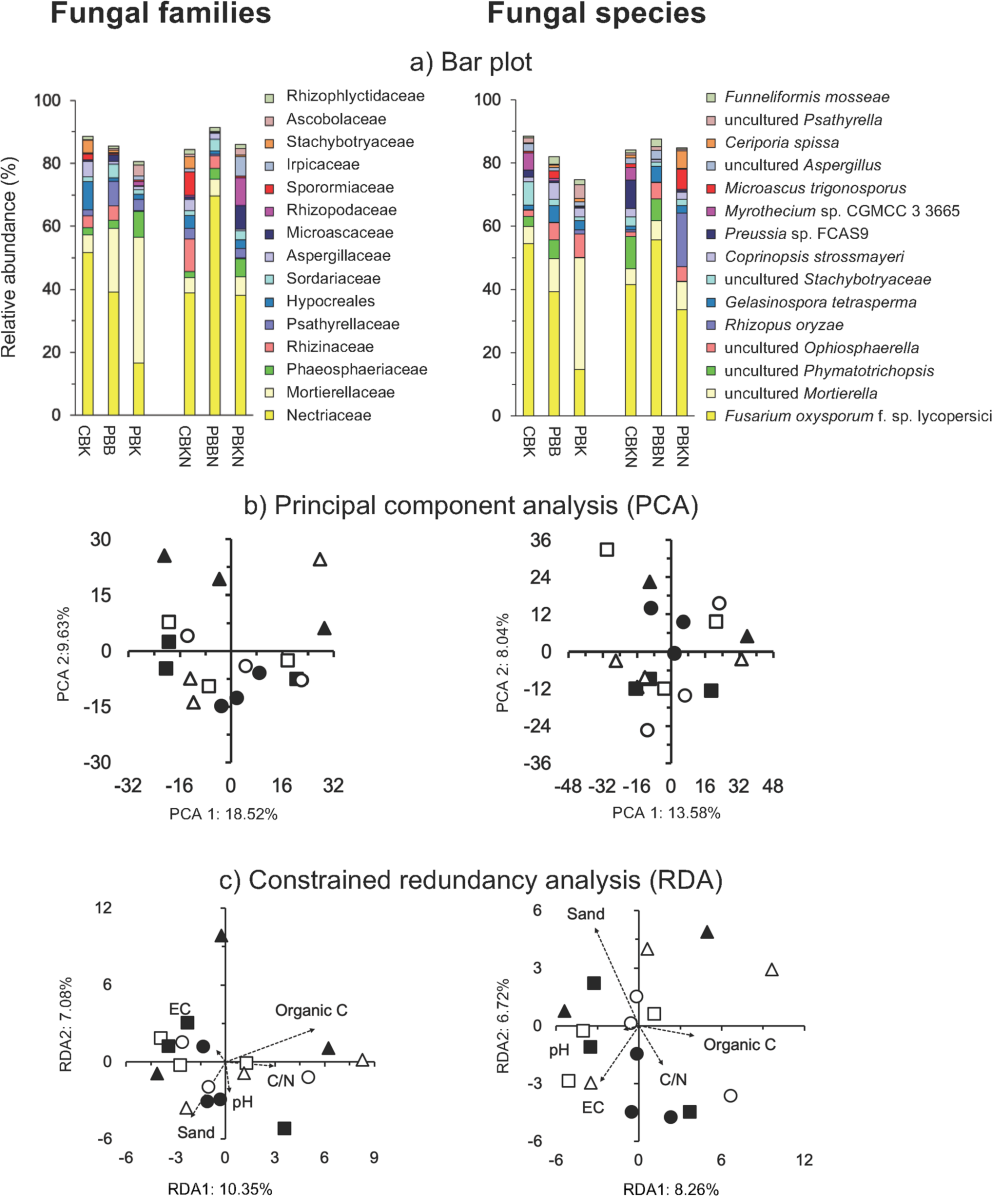
**a** Bar plot with the relative abundance (%) of the 15 most abundant fungal families and species, and **b** principal component analysis (PCA) and **c** constrained redundancy analysis (RDA) with all fungal families and species in soil at the “Campo Experimental Norman E. Borlaug” (CENEB). Abbreviations of the agricultural practices applied at CENEB and the symbols used to represent them are given in the legends to Fig. 1

Nectriaceae (40.0 ± 22.6%) was the most dominant fungal family, while *Fusarium oxysporum* f. sp. *lycopersici* (39.9 ± 22.6%) was the most abundant fungal species (Fig. 2a). The PCA and constrained RDA did not separate the fungal communities in the different treatments and the perMANOVA analysis indicated that none of the agriculture practices studied, i.e., N fertilizer application, burning or tillage affected, the fungal community significantly (Fig. 2b, c; Table S3). The Mantel test showed that soil pH was significantly correlated to the fungal families and species community (*P* < 0.05) (Table S4). Some fungal groups were affected significantly by tillage or burning (*P* < 0.05) and the effect size on the relative abundances of some of them was very large (effect size ≤  − 1.3 or ≥ 1.3) (Fig. S3b; Table S5).

### The Fungal Functional Groups

**Fig. 3:**
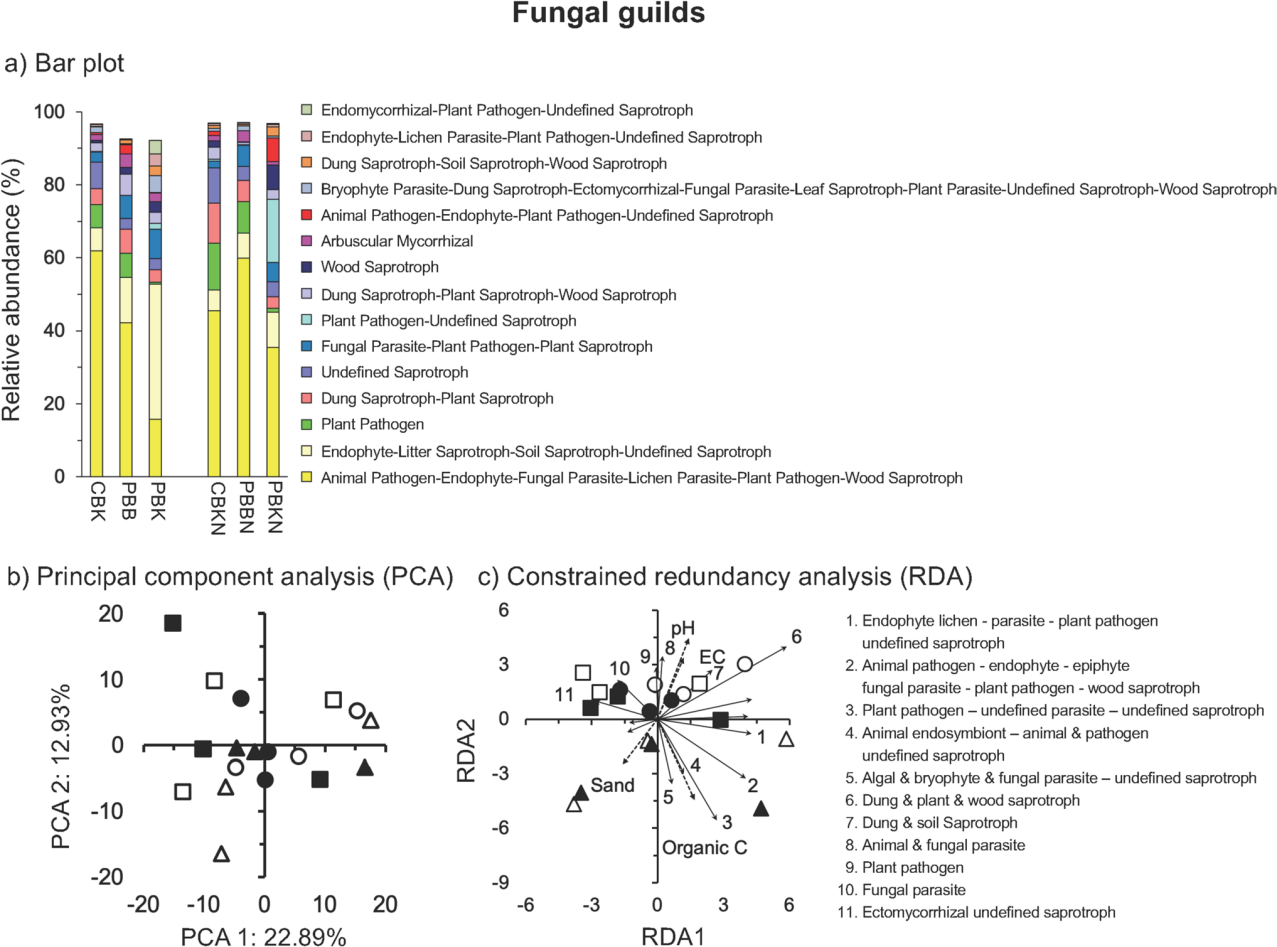
**a** Bar plots with the 15 most abundant fungal guilds (%) as defined by the FUNGuildR package [16], and **b** principal component analysis (PCA) and constrained redundancy analysis (RDA) with all fungal guilds. Abbreviations of the agricultural practices applied at CENEB and the symbols used to represent them are given in the legends to Fig. 1

**Fig. 4:**
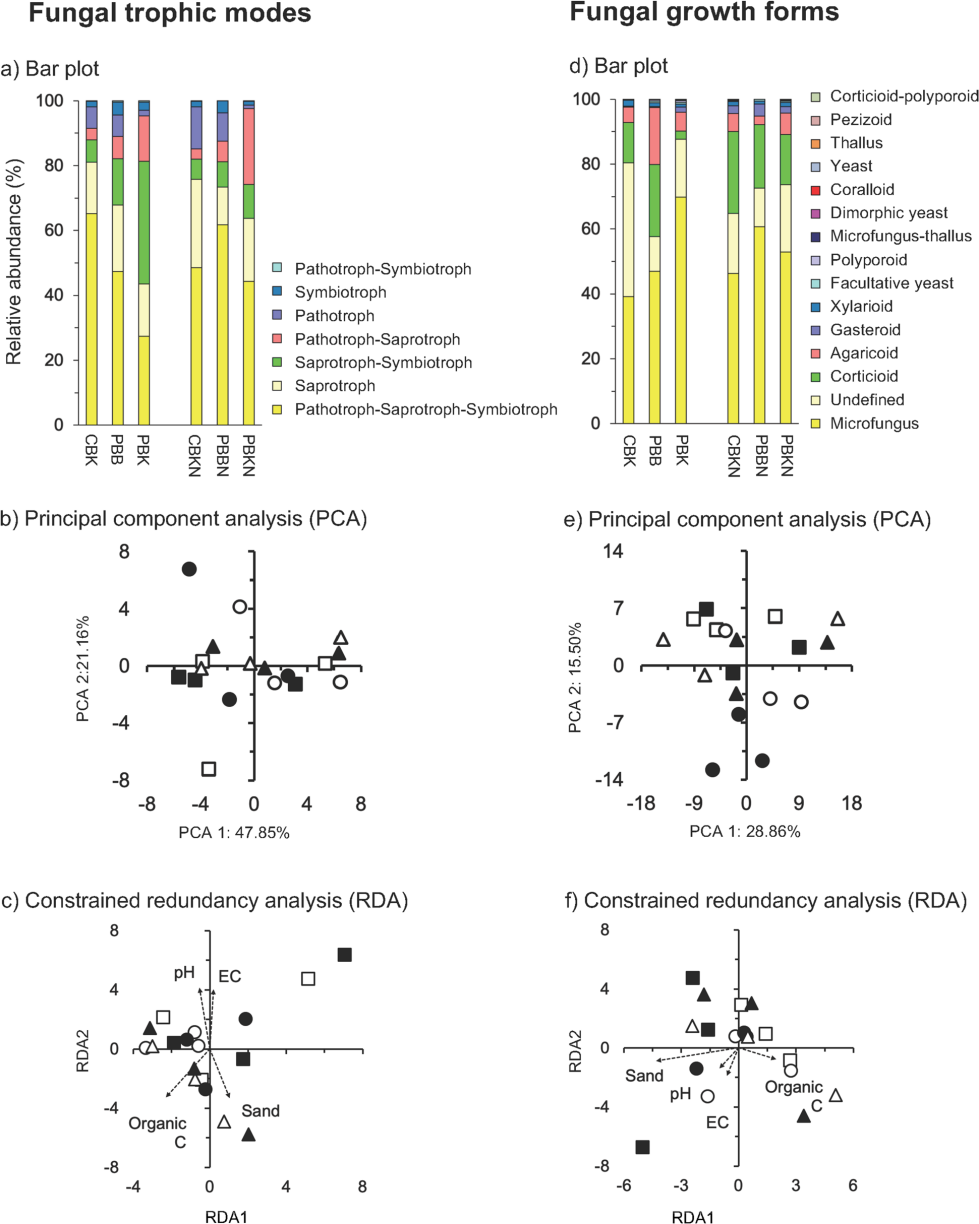
**a** Bar plots with the 15 most abundant the fungal trophic modes (%) as defined by the FUNGuildR package [16]), and **b** principal component analysis (PCA) and **c** constrained redundancy analysis (RDA) with all the fungal trophic modes, **d** Bar plots with the 15 most abundant fungal growth forms (%) as defined by the FUNGuildR package [16], and **e** principal component analysis (PCA) and **f** constrained redundancy analysis (RDA) with all the fungal growth forms. Abbreviations of the agricultural practices applied at CENEB and the symbols used to represent them are given in the legends to Fig. 1

Fungal taxa were classified according to their guild, trophic mode and growth form. The assigned dung and plant saprotroph (18.4%) and plant pathogens (16.0%) were the most abundant guilds, saprothrophic the most abundant trophic mode (52.5%) and microfungus the most abundant growth form. Guilds showed large differences between the treatments, but less for the trophic mode and growth form (Figs. 3a; 4a). Application of N fertilizer, burning of the crop residue or tillage had no significant effect on guilds and the PCA did not separate the guilds in the different treatments (Fig. 3b; Table S3). The fungal community structure in terms of trophic mode and growth form were not affected significantly by N fertilizer application, burning of the crop residue or tillage and the PCA did not separate the fungal communities in the different treatments (Fig. 4b, e; Table S3). The Mantel test showed a significant positive correlation of fungal guild and trophic mode with pH, organic C and sand content, and fungal growth with sand content (*P* < 0.05) (Table S4). The constrained RDA did not separate the fungal trophic mode and growth form in the different treatments at CENEB (Fig. 4c, f). Although the fungal guild abundances were not affected significantly by N fertilizer application, some were affected significantly by burning of the crop residue or tillage (*P* < 0.05) (Table S5). The relative abundance of plant pathogens and undefined saprotrophs increased significantly with tillage, while that of dung-plant and dung-soil saprotroph, and plant pathogens by burning (*P* < 0.05).

### Maize and Wheat Root Rot

Maize and wheat root rot was monitored in the seminal, crown and tiller roots field in the same treatments used in this study from 2008 to 2013 (Fig. S4). Tillage and burning of the crop residue had no significant effect on the scored root rot incidence, although N fertilizer application significantly increased it in 2012, i.e., the year the soil was sampled for this study and considering all years (2008–2013) (Table S7).

## Discussion

### Soil Characteristics

Abbas et al. [25] reported that soil organic C stocks in 10-year wheat-cotton and wheat–maize rotation agricultural practices in semi-arid lands increased 14% (37.2 vs 43.3 Mg ha^−1^) with no-tillage and mulch application compared to soil with conventional tillage and mulch application. In this study, the organic C was 25% higher in the permanent beds than in the conventional tilled beds so burning or incorporating crop residue (tillage) reduced the soil organic matter content compared to the treatment where it was left on the soil surface after 20 years. Burning will eliminate most of the crop residue left on the soil surface and so reduce the amount of organic material that enters the soil, although fire might promote soil organic matter stability thereby increasing C storage in the long-term as its mineralization is reduced [26]. When crop residue is incorporated into the soil with tillage, it comes in direct contact with the soil microorganisms and is quickly mineralized. However, when it remains on the soil surface its decomposition is much slower and it is degraded by abiotic photodegradation and fungal activity while macrofauna activity might, but does not always, contribute to it [27].

In this study, application of N fertilizer increased the total soil N compared to the soil that was left unfertilized. Application of N fertilizer will not only increase plant growth but also its N content. The crop residue with a higher N content that is incorporated or left on the soil surface will increase the soil organic N content. Additionally, the soil microorganisms might immobilize part of the amended N fertilizer as the crop residues have a high C-to-N ratio. This reduces possible losses of mineral N and so further increases the soil organic N content.

Arunrat et al. [28] found that even after a low intensity fire in rotational shifting cultivation in Northern Thailand the EC and pH increased in soil as found in this study. Blevins et al. [29] contributed this increase to the oxidation of surface applied N fertilizer and applying lime on the soil surface has been shown to neutralize this acidification under no-tillage. In this study, the opposite was found. The straw cover in permanent beds with residue retained reduced evaporation that brings salts to the surface. As such, the EC tends to be higher when the crop residue is burned or incorporated compared to where it is left on the surface as evaporation is higher in the first and lower in the latter.

### The Protist Community

Protists are generally considered all eukaryotes except plants, fungi and animals. Leff et al. [30] reported that the protist community in a grassland soil was dominated by Rhizaria (26%), Amoebozoa (25%), Alveolata (22%) and Stramenopiles (16%). In this study, the protists were dominated by Alveolata and Amoebozoa. The members of Alveolata (SAR) have diverse forms of nutrition, i.e., intracellular parasitism, photoautotrophy and predation [31]. They were represented by only two subgroups: Ciliophora, the most abundant subgroup, characterized by cilia in at least one of their life stages and by their nuclear dimorphism and Protalveolata, which lost photosynthesis and retained feeding on eukaryotic prey [32]. Amoebozoa includes amoeboid and flagellate organisms with single cells that have adopted many lifestyles and can live in a wide range of environments [33].

*Acanthamoeba* (Amoebozoa), the most abundant protists genus in this study, is distributed widely in soil and some members of this genus are pathogenic in humans [34]. Stichotrichia, a subclass of ciliates that contains 18 families and 204 genera (https://eol.org/pages/2908969), included the most abundant protist species in this study, i.e., *Stichotrichia* sp. 1 bAcq1. Phylotypes that belong to the *Colpoda*, the second most abundant protist genus in this study, are bacterivorous protozoan and might alter the microbial community structure in soil as they target specific bacterial groups [35]. *Dictyamoeba* has been found in a garden soil sample with moss and lichens in Wales (UK) [36], while some groups, such as Ochrophyta (a group of mostly photosynthetic heterokonts) are mostly aquatic. The latter might have entered the soil at CENEB with the irrigation water.

Although protists play an important role in soil, the factors controlling their distribution and how they are affected by environmental conditions are still poorly understood [37]. Bates et al. [38] found that protist community structures were controlled by climatic conditions; mostly precipitation. In drier conditions, the abundances of active protists can drop dramatically as they form cysts, i.e., a mechanism for survival in dry periods. Ma et al. [39] reported that tillage had a significant effect on the protistan community structure, while Hu et al. [40] found that application of chemical fertilizers had a limited effect on the protistan alpha diversity and the community, although manure had a clear effect. Wang et al. [41] reported that protist increase litter decomposition, but it was species-specific and some might even reduce it. In this study, the protists community structure was not affected by agricultural practices and the relative abundance of only a limited number of protists was. *Stichotrichia* sp. bAcq1 was enriched in the N fertilized soil and *Ischnamoeba* sp FN352 in conventional tilled beds with the crop residue incorporated. Members of *Ischnamoeba,* are non-marine and strict bacterivorous [36], but why they were enriched in the tilled soil with incorporation of crop residue remains to be investigated. However, it has to be stressed that the primers used were biased towards fungi, so we might have missed some part of the protist community as their phylogenetic diversity makes it difficult to have a universal primer for them [42].

### The Fungal Community

Fungi, members of Nucletmycea, was the most abundant microscopic eukaryotes group in this study. Four different fungal phyla were detected in the arable soil at CENEB, i.e., Ascomycota, Chytridiomycota, Basidiomycota and Glomeromycota. Ascomycota are often dominant in arable soils [43], as in this study. Chytridiomycota are saprotrophic, i.e., chemoheterotrophic extracellular digestion of decayed (dead or waste) organic matter, which might explain their high relative abundance in the arable soil. Basidiomycota contain most of the ectomycorrhizal fungi and their relative abundance is normally lower in an arable than in a forest soil [44]. The relative abundance of the Glomeromycota (most of its members form arbuscular mycorrhizas) was low although the soil was sampled when wheat was still growing [43].

Members of Nectriaceae (Hypocreales, Ascomycetes) that include human and plant pathogens, and numerous species with commercial use as biocontrol agents and biodegraders were the most abundant fungal family found in the studied soils. *Fusarium* was the most abundant fungal genus and is saprophytic (defined as: *“feeding, absorbing or growing upon decaying organic matter”* (https://www.biologyonline.com/dictionary/saprophytic). It is commonly found in soil and contains many different opportunistic plant pathogens [45]. *Fusarium oxysporum* f sp. *Lycopersici* was the most abundant species and is a causal agent of vascular wilt disease of tomato.

Agricultural practices have often been found to alter the fungal community in soil, rhizosphere and roots of cultivated crops. Sommermann et al. [46] investigated the effect of conventional vs. conservation tillage, intensive vs. extensive fertilization and different pre-crops (maize vs. rapeseed) on the fungal communities in a long-term crop rotation field trial established in 1992 in Central Germany. They found a significant effect of tillage and pre-crop on the fungal community structures, but not fertilization. Garnica et al. [47] reported that the fungal community in roots of winter wheat (*Triticum aestivum* L.) grown in organic farming systems differed and were richer than those of wheat roots grown in conventional practices. Essel et al. [48] found that no-till and residue retention practices influenced fungal species diversity through improved soil chemical properties, which have potential to affect the habitat and activity of soil microbes. Ma et al. [39] reported that tillage had a significant effect on the fungal community structure. In this study, none of the studied factors, i.e., tillage, inorganic N fertilizer application or crop residue management had a significant effect on the fungal community structure. Klaubauf et al. [49] also found no differences in fungal diversity when comparing a grassland with an arable soil, while Ding et al. [50] found that fungal diversity decreased when inorganic fertilizer was to soil. This would suggest that the interactions between soil characteristics, climate conditions and plants grown might determine if the agricultural practices, which themselves might differ from experiment to experiment, alter the fungal community in an agroecosystem.

Soil pH is a major driver of fungal diversity in arable soil under long-term fertilization regimes and often has the most significant effect on fungal communities [51]. Although the pH and other soil characteristics, e.g., soil N and C content, were different between the different treatments at CENEB the fungal diversity was not affected by agricultural practices.

The fungal community structure was not altered by agricultural practices, but some fungal groups were affected significantly by tillage and burning, but not by N fertilizer application. An uncultured member of *Phymatotrichopsi*, a genus of ascomycete fungi in the family Rhizinaceae, and *Nowakowskiella elegans* (NCBI:txid10988) a species of chytrid in the family Nowakowskiellaceae (order Cladochytriales) were enriched when crop residue was incorporated compared to where the crop residue was left on the soil surface. The genus *Phymatotrichopsi* contains *Phymatotrichopsis omnivora*, the causal agent of Phymatotrichum root rot of more than 2,000 dicotyledonous plant species [52]. The relative abundance of *Aspergillus niger* decreased when crop residue was burned while *Gelasinospora tetrasperma* also called *Neurospora tetraspora* (NCBI:txid94610) was enriched*. Aspergillus niger* is well-known to participate in the degradation of organic material [53], so burning of crop residue in the field might reduce its abundance. *Gelasinospora tetrasperma*, a homothallic, ascosporic filamentous fungus having 4-spored asci, was isolated from a non-rhizosphere soil sample collected from a grapevine plantation in the village of El-Khawaled [54]. Why it was enriched when the crop residue was burned is difficult to explain, but its capacity to grow on wood might have given it the capacity to degrade the more resistant organic material that remained after burning of the crop residue.

### Fungal Functional Groups

Crop residue management might affect fungal guilds and plant residues are important in defining the fungal community structure [55]. Plant pathogens might survive in crop residue that is left on the soil surface as the soil temperature is lower, moisture content is higher and the residue might serve as a refuge for the pathogen. In this study, the relative abundance of plant pathogens (mostly *Phymatotrichopsis*) was lower when crop residue was left on the soil than when burned or incorporated. *Phymatotrichopsis omnivore* causes Phymatotrichum (cotton) root rot in more than 2,000 dicotyledonous plant species and is found mostly in south‐western USA and northern Mexico, Libya and Venezuela and possibly parts of central Asia [52]. It prefers lightly alkaline soils and higher temperatures and is affected by soil moisture content [56]. The disease is difficult to control but deep ploughing and incorporation of crop residue seems to reduce the disease (https://plantdiseasehandbook.tamu.edu/problems-treatments/problems-affecting-multiple-crops/cotton-root-rot/). Why the relative abundance of this pathogenic fungus was lower when the crop residue was left on the soil surface in this field experiment is difficult to explain. It must be stressed, however, that the crop residue left on the soil surface was not analyzed for fungi. The crop residue left on the soil surface might have served as a reservoir for *Phymatotrichopsis omnivore.* However, the increase or decrease in the relative abundance of a pathogen does not necessarily means that its pathogenicity (and/or its virulence (the severity, harmfulness or degree of damage of the disease) are affected in the same way. In this study, changes in the relative abundance of plant pathogens (mostly *Phymatotrichopsis*) due to agricultural practices was not matched by disease, i.e., root rot, in the crops [57].

Tillage is known to alter the fungal community structure [58]. Orrù et al. [15] found a higher diversity of saprotrophic fungi in soil with low disturbances. Gu et al. [59] reported that arbuscular mycorrhizal fungal community was also affected by tillage, but found that residue management had a more limited effect on them. The increase in the relative abundance of arbuscular mycorrhizal fungi in no-till system is due to increased aggregate stability. In this study, the relative abundance of arbuscular mycorrhizal did not increase. Different soil characteristics, climate conditions, crop rotations, agricultural practices and their interactions will affect how a certain factor might affect fungal groups, guilds and their activity. For instance, Tedersoo et al. [60] based on 365 global soil samples from different ecosystems concluded that “*climatic factors, followed by edaphic and spatial patterning, were the best predictors of soil fungal richness and community composition at the global scale*”.

The relative abundance of the guild “dung saprotrophs—plant saprotrophs”, mostly *Gelasinospora tetrasperma* and *Preussia* sp. CCF3831, was higher in the treatments where the crop residue was incorporated or burned compared to where it was left on the soil surface. *Gelasinospora tetrasperma* is an ascomycete fungus that belongs to the Sordariales which can be found in different environments, such as litter, dung, soil and burned vegetation with most of its members considered saprothrops although some are plant endophytes and might be even pathogens (https://mycocosm.jgi.doe.gov/Gelte1/Gelte1.home.html). Some members of Sordariales are thermophilic which might explain their high relative abundance in soil where the crop residue was burned while the incorporated crop residue provided them with an abundant substrate for growth. *Preussia* belongs to the Sporormiaceae (Pleosporales) and grows on dung or in the soil and some of its members show antimicrobial activity [61]. As such, these coprophilous fungi might be of interest for their production of secondary metabolites that might help in plant pathogens control.

The relative abundance of the guild “dung saprotrophs—soil saprotrophs”, mostly an uncultured Ascodesmidaceae*,* i.e., ascomycete sp. BEG01 (Pezizales), was higher in the treatments where the crop residue was burned compared to where it was left on the soil surface. Ascodesmidaceae*,* such as *Ascodesmis,* are also coprophilous and grow on animal dung [62]. Their spores are hard and when eaten by animals, mostly herbivores, pass through the digestive track, grow on their feces and form spores that are eaten again. They might have entered the soil through the irrigation water or through the presence of small rodents (mice and rats although their numbers are generally small) that might have eaten the spores.

## Conclusion

Burning the crop residue significantly reduced the soil organic C content and increased the pH and electrolytic conductivity (EC) compared to where it was left on the soil surface of the permanent beds and the organic C content in the latter was significantly higher than when it was incorporated, but the pH and EC was lower. The fungal and protist community structures and fungal assigned guilds were not affected by tillage, N fertilizer application or burning. However, some fungal groups and fungal assigned guilds were affected significantly by tillage and burning, but less so by N fertilizer application. Plant pathogens (mostly *Phymatotrichopsi* with *P. omnivora* the causal agent of Phymatotrichum root rot of more than 2000 dicotyledonous plant species), and dung-plant and undefined saprotrophs were significantly reduced by tillage, while dung-plant and dung-soil saprotroph, and plant pathogens by burning. Why the relative abundance of plant pathogens was lower when the crop residue was left on the soil surface in this field experiment is difficult to explain but it must be stressed that the crop residue left on the soil surface was not included in the analysis so it might have served as a reservoir for *Phymatotrichopsis omnivore.* It might be interesting in future, to use shotgun methanogenics which might allow a more in depth study of the microscopic eukaryotes; especially protists as primers are biased against them.

## Supplementary Information

Below is the link to the electronic supplementary material.

**Fig. S1** Soil sampling procedure and treatments applied to the different soils. Supplementary file1 (TIFF 301 kb)

**Fig. S2** Boxplots with the Hill numbers at q = 0, q = 1 and q = 2 of a) the protist and fungal species. Treatments used from the “Campo Experimental Norman E. Borlaug” (CENEB) in this study were CBK: conventional tilled beds, i.e., beds are formed after each crop, all crop residues kept and incorporated by conventional tillage, no N fertilizer application, CBKN: the same as CBK but with N fertilizer application at 300 kg urea-N ha^−1^ y^−1^, PBB: permanent beds are reshaped as needed, wheat and maize straw burned, no N fertilizer application, PBBN: the same as PBB but with N fertilizer application at 300 kg urea-N ha^−1^ y^−1^, PBK: permanent beds are reshaped as needed, both wheat and maize straw chopped and left in place, no fertilizer application, PBKN: the same as PBK but with fertilizer application at 300 kg urea-N ha^−1^ y^−1^. Df-1, Degrees of freedom 1. Supplementary file2 (TIFF 334 kb)

**Fig. S3** Volcano plot comparing the relative abundance of a) protist and b) fungal species in the unfertilized (CBK - PBB - PBK) vs the fertilized soil (CBKN - PBBN - PBKN), the tilled beds (CBK - CBKN) vs the untilled beds (PBK - PBKN) and the soil with burned crop residues (PBB - PBBN) vs the crop residue left on the soil surface (PBK - PBKN). The expected p value of the Kruskal-Wallis test is given in the y-axis and the effect size is given in the x-axis [35]. The effect size, which is defined as the difference between groups divided by the maximum dispersion within group A or B, was calculated with the ALDEx2 package using the aldex.ttest argument. A negative value indicates that the relative abundance of the microbial group was higher in the first mentioned soil than in the second mentioned one and a positive value the opposite. Vertical lines indicate large effects size (≤ − 0.8, ≥ 0.8) and very large effect sizes (≤ − 1.3, ≥ 1.3) [63]. Abbreviations of the agricultural practices applied at CENEB are given in the legends to Fig. S2. Supplementary file3 (TIFF 656 kb)

**Fig. S4** Maize and wheat root rot in the a) seminal, b) crown and c) tiller roots field in the CBK, PBB, PBK, CBKN, PBBN and PBKN treatments fat CENEB from 2008 to 2013. Abbreviations of the agricultural practices applied at CENEB are given in the legends to Fig. S2. Supplementary file4 (TIFF 399 kb)

Supplementary file5 (DOCX 20 kb)

Supplementary file6 (DOCX 23 kb)

Supplementary file7 (DOCX 19 kb)

Supplementary file8 (DOCX 20 kb)

Supplementary file8 (DOCX 22 kb)

## Data Availability

Data will be made available on request.
