# Supplementary figures and images for: The Fungal and Protist Community as Affected by Tillage, Crop Residue Burning and N Fertilizer Application
Source: Curr Microbiol. 2025 Feb 19;82(4):144. doi: 10.1007/s00284-025-04112-5 (PMC11839885; doi:10.1007/s00284-025-04112-5)

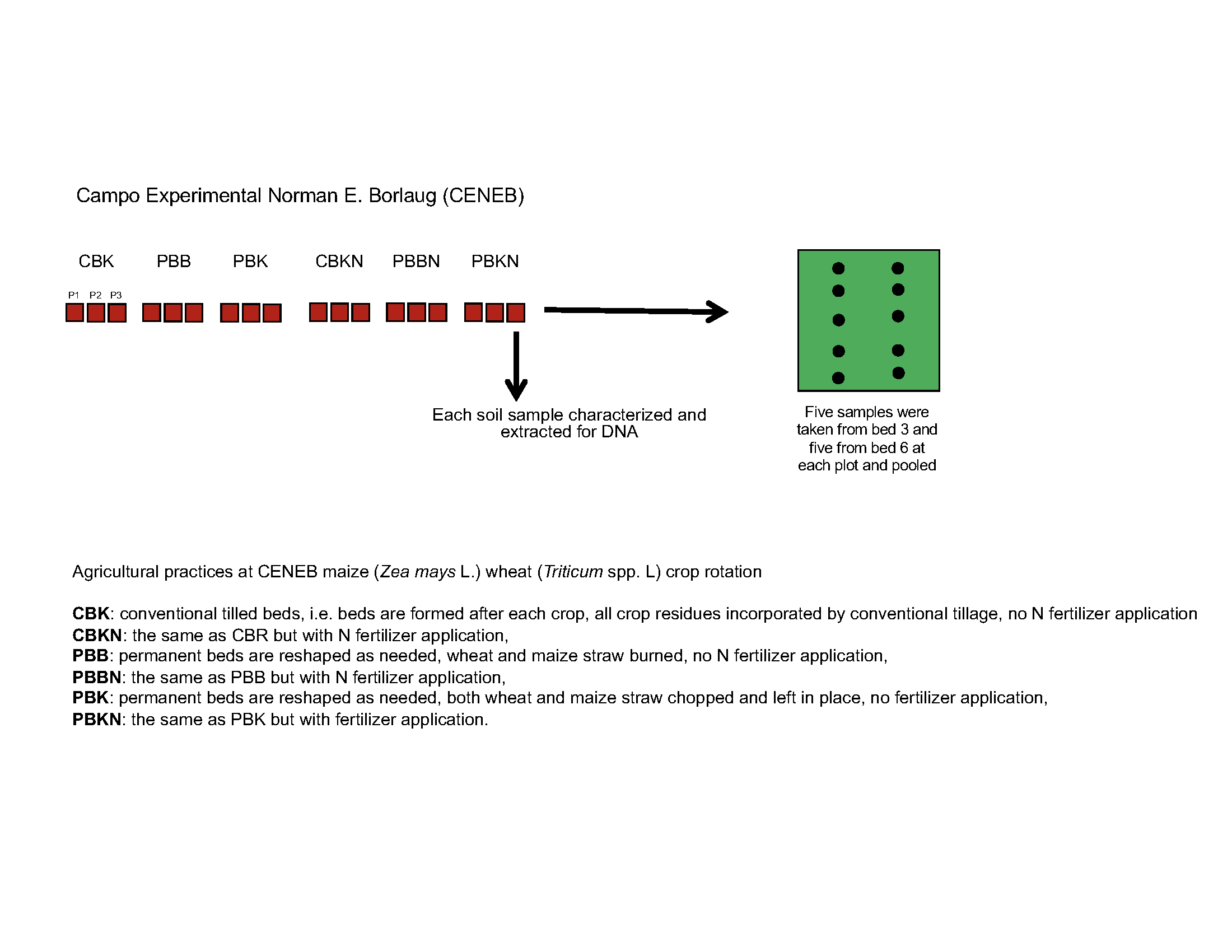

Supplement: Supplementary file 1 — Fig. S1 Soil sampling procedure and treatments applied to the different soils. Supplementary file1 (TIFF 301 kb) [file 284_2025_4112_MOESM1_ESM.tiff]

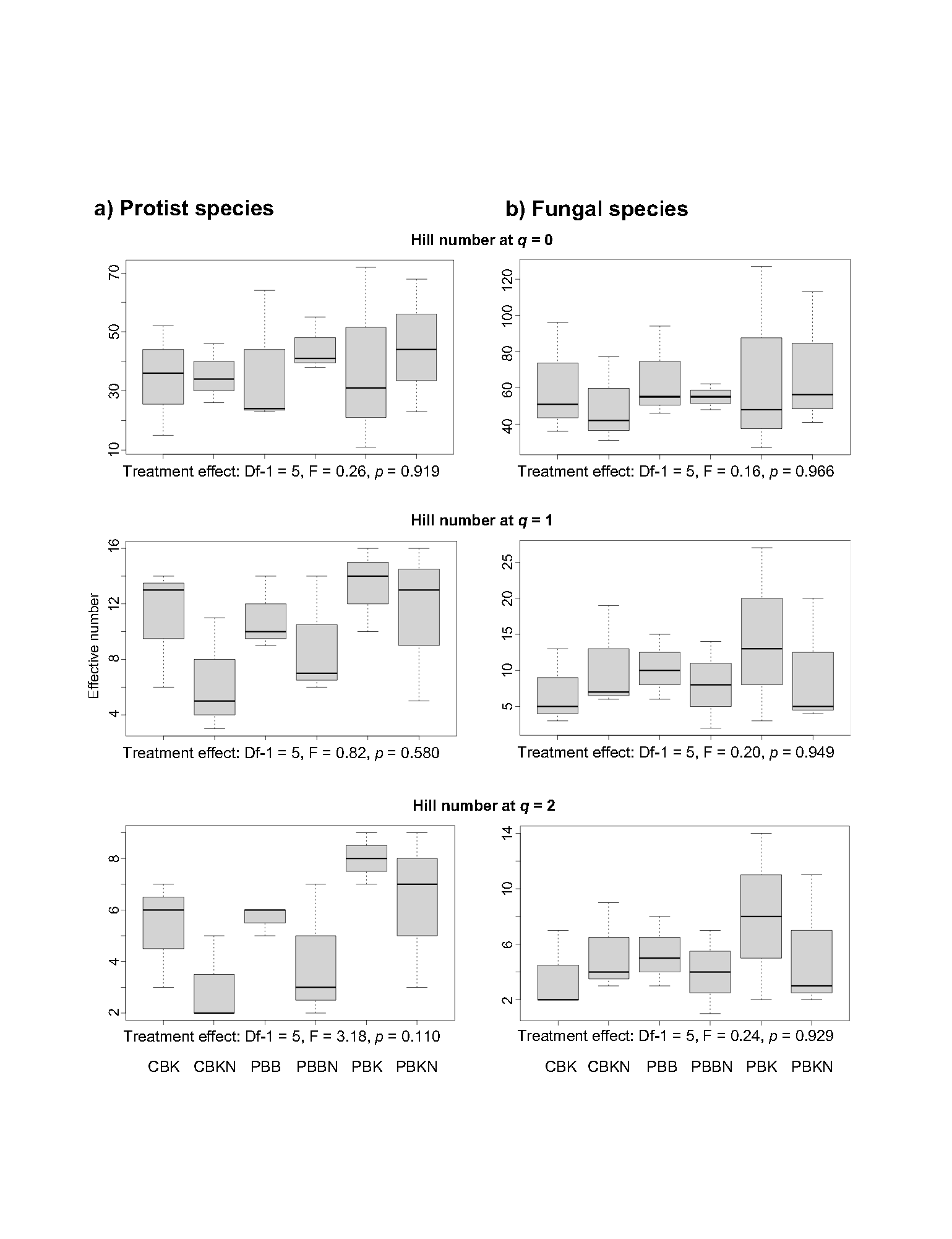

Supplement: Supplementary file 2 — Fig. S2 Boxplots with the Hill numbers at q = 0, q = 1 and q = 2 of a) the protist and fungal species. Treatments used from the “Campo Experimental Norman E. Borlaug” (CENEB) in this study were CBK: conventional tilled beds, i.e., beds are formed after each crop, all crop residues kept and incorporated by conventional tillage, no N fertilizer application, CBKN: the same as CBK but with N fertilizer application at 300 kg urea-N ha−1 y−1, PBB: permanent beds are reshaped as needed, wheat and maize straw burned, no N fertilizer application, PBBN: the same as PBB but with N fertilizer application at 300 kg urea-N ha−1 y−1, PBK: permanent beds are reshaped as needed, both wheat and maize straw chopped and left in place, no fertilizer application, PBKN: the same as PBK but with fertilizer application at 300 kg urea-N ha−1 y−1. Df-1, Degrees of freedom 1. Supplementary file2 (TIFF 334 kb) [file 284_2025_4112_MOESM2_ESM.tiff]

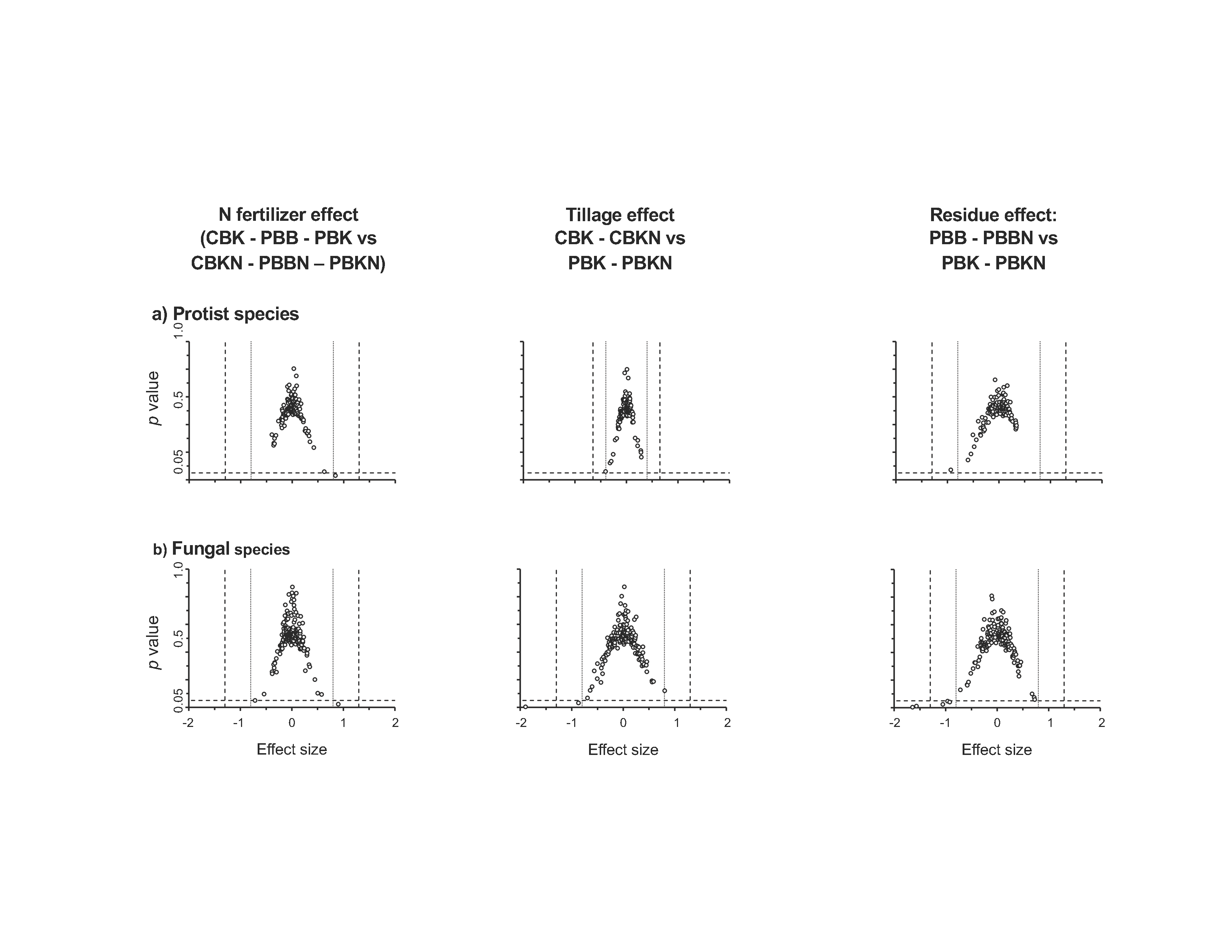

Supplement: Supplementary file 3 — Fig. S3 Volcano plot comparing the relative abundance of a) protist and b) fungal species in the unfertilized (CBK - PBB - PBK) vs the fertilized soil (CBKN - PBBN - PBKN), the tilled beds (CBK - CBKN) vs the untilled beds (PBK - PBKN) and the soil with burned crop residues (PBB - PBBN) vs the crop residue left on the soil surface (PBK - PBKN). The expected p value of the Kruskal-Wallis test is given in the y-axis and the effect size is given in the x-axis [35]. The effect size, which is defined as the difference between groups divided by the maximum dispersion within group A or B, was calculated with the ALDEx2 package using the aldex.ttest argument. A negative value indicates that the relative abundance of the microbial group was higher in the first mentioned soil than in the second mentioned one and a positive value the opposite. Vertical lines indicate large effects size (≤ − 0.8, ≥ 0.8) and very large effect sizes (≤ − 1.3, ≥ 1.3) [63]. Abbreviations of the agricultural practices applied at CENEB are given in the legends to Fig. S2. Supplementary file3 (TIFF 656 kb) [file 284_2025_4112_MOESM3_ESM.tiff]

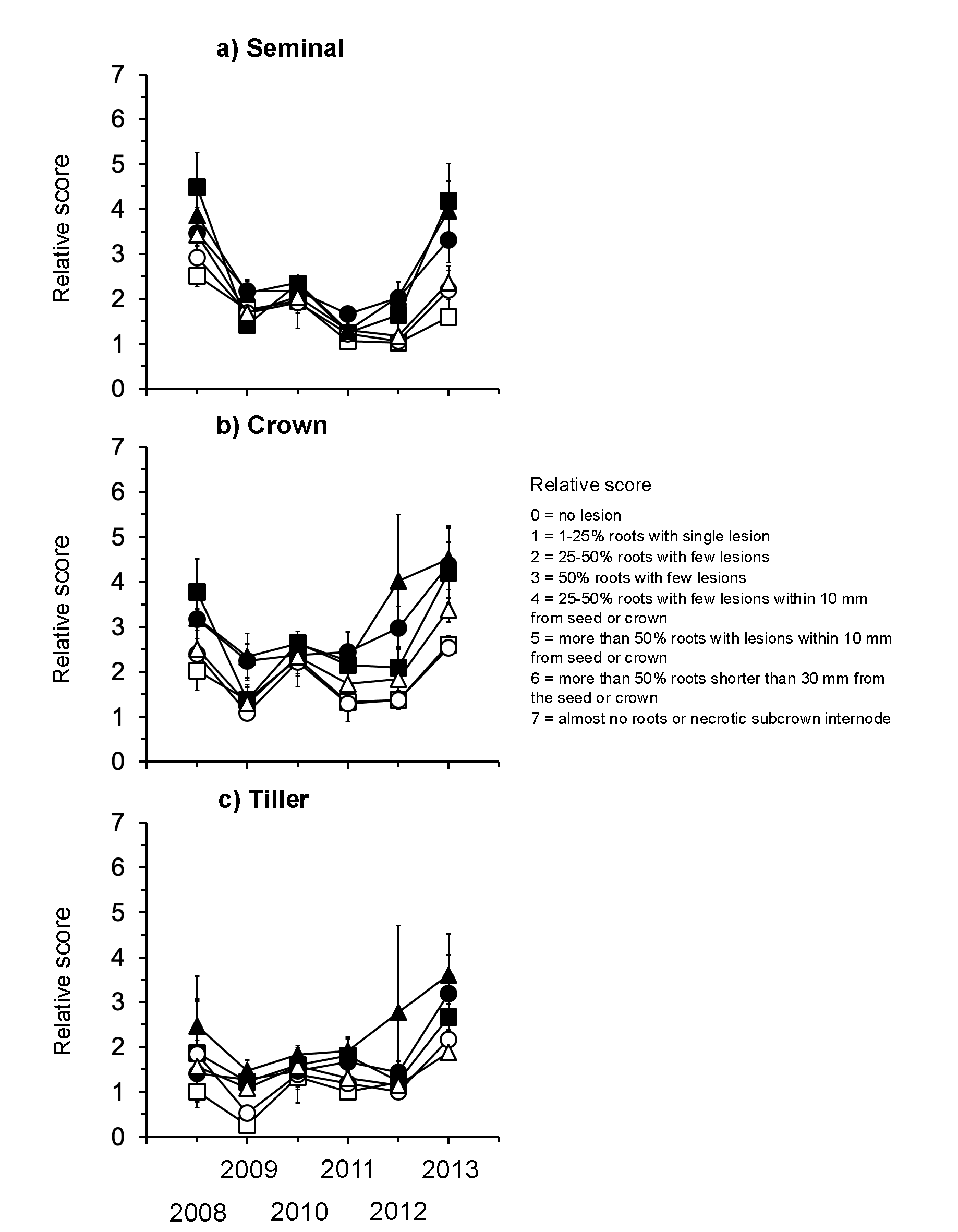

Supplement: Supplementary file 4 — Fig. S4 Maize and wheat root rot in the a) seminal, b) crown and c) tiller roots field in the CBK, PBB, PBK, CBKN, PBBN and PBKN treatments fat CENEB from 2008 to 2013. Abbreviations of the agricultural practices applied at CENEB are given in the legends to Fig. S2. Supplementary file4 (TIFF 399 kb) [file 284_2025_4112_MOESM4_ESM.tiff]
