# Supplementary material for: The Fungal and Protist Community as Affected by Tillage, Crop Residue Burning and N Fertilizer Application
Source: Curr Microbiol. 2025 Feb 19;82(4):144. doi: 10.1007/s00284-025-04112-5 (PMC11839885; doi:10.1007/s00284-025-04112-5)
Supplement: Supplementary file 5 — Supplementary file5 (DOCX 20 kb) [file 284_2025_4112_MOESM5_ESM.docx]

**Table S3** Permutational multivariate analysis of variance (perMANOVA) to determine the effect of burning (permanent beds (PB) crop residue burned vs PB crop residue retained), N fertilizer (crop unfertilized (0 kg urea-N ha^-1^) vs crop fertilized with 300 kg urea N ha^-1^) and tillage (PB with crop residue retained vs conventional tilled beds with crop residue incorporated) on the microscopic eukaryotes, protists community, fungal and assigned guilds structure in soil at CIMMYT’s Norman E. Borlaug (CENEB) experimental station near Ciudad Obregón (Sonora, Mexico).

| ⎯⎯⎯⎯⎯⎯⎯⎯⎯⎯⎯⎯⎯⎯⎯⎯⎯⎯⎯⎯⎯⎯⎯⎯⎯⎯⎯⎯⎯⎯⎯⎯⎯⎯⎯⎯ | | | | | |
| --- | --- | --- | --- | --- | --- |
| Microorganisms | Taxonomic level | Factor | F value | *p* value |  |
| ⎯⎯⎯⎯⎯⎯⎯⎯⎯⎯⎯⎯⎯⎯⎯⎯⎯⎯⎯⎯⎯⎯⎯⎯⎯⎯⎯⎯⎯⎯⎯⎯⎯⎯⎯⎯ | | | | | |
| Microscopic eukaryotes | Level 3 | Burning ^a^ | 0.89 | 0.520 |  |
|  |  | N fertilizer | 0.59 | 0.953 |  |
|  |  | Tillage | 1.26 | 0.213 |  |
| Protist | Species | Burning | 0.87 | 0.749 |  |
|  |  | N fertilizer | 1.10 | 0.258 |  |
|  |  | Tillage | 0.86 | 0.846 |  |
| Fungi | Families | Burning | 1.07 | 0.313 |  |
|  |  | N fertilizer | 0.84 | 0.756 |  |
|  |  | Tillage | 1.05 | 0.304 |  |
|  | Species | Burning | 1.13 | 0.209 |  |
|  |  | N fertilizer | 1.07 | 0.269 |  |
|  |  | Tillage | 1.08 | 0.267 |  |
| Assigned ^b^ | Taxon | Burning | 0.95 | 0.559 |  |
|  |  | N fertilizer | 0.74 | 0.926 |  |
|  |  | Tillage | 1.00 | 0.412 |  |
|  | Guilds | Burning | 1.18 | 0.215 |  |
|  |  | N fertilizer | 1.16 | 0.243 |  |
|  |  | Tillage | 1.11 | 0.286 |  |
|  | Trophic mode | Burning | 0.74 | 0.553 |  |
|  |  | N fertilizer | 1.40 | 0.229 |  |
|  |  | Tillage | 1.29 | 0.280 |  |
|  | Growth form | Burning | 0.64 | 0.838 |  |
|  |  | N fertilizer | 0.65 | 0.810 |  |
|  |  | Tillage | 0.99 | 0.420 |  |
| ⎯⎯⎯⎯⎯⎯⎯⎯⎯⎯⎯⎯⎯⎯⎯⎯⎯⎯⎯⎯⎯⎯⎯⎯⎯⎯⎯⎯⎯⎯⎯⎯⎯⎯⎯⎯⎯ | | | | | |

^a^ The degree of freedom was 1 and the number of replicates *s* = 6, ^b^ Guilds assigned using the FUNGuildR package (version 0.2.0.9000) based on Nguyen et al. [8].

| ⎯⎯⎯⎯⎯⎯⎯⎯⎯⎯⎯⎯⎯⎯⎯⎯⎯⎯⎯⎯⎯⎯⎯⎯⎯⎯⎯⎯⎯⎯⎯⎯⎯⎯⎯⎯⎯ |
| --- |
