# Supplementary material for: The Fungal and Protist Community as Affected by Tillage, Crop Residue Burning and N Fertilizer Application
Source: Curr Microbiol. 2025 Feb 19;82(4):144. doi: 10.1007/s00284-025-04112-5 (PMC11839885; doi:10.1007/s00284-025-04112-5)
Supplement: Supplementary file 6 — Supplementary file6 (DOCX 23 kb) [file 284_2025_4112_MOESM6_ESM.docx]

**Table S4** Mantel test between soil characteristics, and microscopic eukaryotes, protist species, fungal families and species, and FunGuild grow, guild and trophic modes in soil at the “Campo Experimental Norman E. Borlaug” (CENEB).

| ————————————————————————————————————————————————————————————————— | | | | | | | | | | | | | | | | |  |
| --- | --- | --- | --- | --- | --- | --- | --- | --- | --- | --- | --- | --- | --- | --- | --- | --- | --- |
|  | pH | | EC ^a^ | | Organic C | | Total N | | C/N | | Clay | | Sand | | Loam | |  |
|  | —————— | | —————— | | —————— | | —————— | | —————— | | —————— | | —————— | | —————— | |  |
| Microorganisms | r ^b^ | *p* ^c^ | r | *p* | r | *p* | r | *p* | r | *p* | r | *p* | r | *p* | r | *p* |  |
| ————————————————————————————————————————————————————————————————— | | | | | | | | | | | | | | | | |  |
| Microscopic eukaryotes | 0.15 | 0.121 | 0.14 | 0.144 | 0.08 | 0.215 | 0.02 | 0.383 | -0.02 | 0.488 | -0.10 | 0.771 | 0.03 | 0.359 | -0.17 | 0.968 |  |
| Protist species | 0.13 | 0.139 | 0.13 | 0.130 | 0.11 | 0.156 | 0.03 | 0.368 | 0.02 | 0.396 | 0.05 | 0.280 | 0.07 | 0.277 | 0.01 | 0.394 |  |
| Fungal families | 0.24 | 0.041^*^ ^d^ | 0.12 | 0.194 | 0.17 | 0.113 | -0.11 | 0.796 | 0.17 | 0.119 | -0.15 | 0.868 | 0.18 | 0.137 | -0.24 | 0.984 |  |
| Fungal species | 0.24 | 0.041^*^ | 0.12 | 0.181 | 0.16 | 0.109 | -0.11 | 0.788 | 0.17 | 0.117 | -0.15 | 0.881 | 0.20 | 0.117 | -0.24 | 0.986 |  |
| FunGuild grow | 0.20 | 0.055 | 0.16 | 0.106 | 0.18 | 0.082 | -0.11 | 0.806 | 0.05 | 0.315 | -0.02 | 0.498 | 0.31 | 0.022^*^ | -0.05 | 0.594 |  |
| FunGuild guild | 0.31 | 0.016^*^ | 0.17 | 0.112 | 0.26 | 0.033^*^ | -0.18 | 0.932 | 0.15 | 0.160 | -0.08 | 0.681 | 0.31 | 0.037^*^ | -0.14 | 0.846 |  |
| FunGuild trophic mode | 0.28 | 0.026^*^ | 0.17 | 0.112 | 0.26 | 0.033^*^ | -0.17 | 0.932 | 0.16 | 0.127 | -0.11 | 0.778 | 0.35 | 0.016^*^ | -0.14 | 0.842 |  |
| ————————————————————————————————————————————————————————————————— | | | | | | | | | | | | | | | | |  |
| ^a^ EC: Electrolytic conductivity, ^b^ r: Mantel statistic, ^c^ *p*: p value, ^d^ ^*^ *p* ≤ 0.05. | | | | | | | | | | | | | | | | | |
