# Supplementary material for: The Fungal and Protist Community as Affected by Tillage, Crop Residue Burning and N Fertilizer Application
Source: Curr Microbiol. 2025 Feb 19;82(4):144. doi: 10.1007/s00284-025-04112-5 (PMC11839885; doi:10.1007/s00284-025-04112-5)
Supplement: Supplementary file 7 — Supplementary file7 (DOCX 19 kb) [file 284_2025_4112_MOESM7_ESM.docx]

**Table S5** Effect of N fertilizer application, tillage and burning on the relative abundance of protists groups and fungal species, and guilds assigned using the FUNGuildR package (version 0.2.0.9000) based on Nguyen et al. [8]. Only groups with an effect size ≥ 0.8 or ≤ -0.8 and a significant effect at *p* ≤ 0.05 are given.

| ———————————————————————————————————————————————————————— | |
| --- | --- |
| Fertilizer effect: the relative abundance of the microbial group in the unfertilized (CBK, PBB, PBK) ^a^ versus that in the fertilized soil (CBKN, PBBN, PBKN) ^a^ | |
| ———————————————————————————————————————————————————————— | |
| Protist: *Stichotrichia* sp bAcq1 (0.8 ^b^, 0.031 ^c^ ^*^ ^d^)  Fungi: Uncultured fungus (0.9, 0.024^*^) | |
| ———————————————————————————————————————————————————————— | |
| Tillage effect: the relative abundance of the microbial group in soil with conventional tilled beds (CBK, CBKN) versus permanent beds (PBK, PBKN) | |
| ———————————————————————————————————————————————————————— | |
| Protist: *Ischnamoeba* sp FN352 (-0.8, 0.043^*^), Holozoa (1.1, 0.021^*^),  Fungi: uncultured *Phymatotrichopsis* (-1.9, 0.004^**^), Aspergillaceae (-1.6, 0.010^**^), Rhizinaceae (-1.5, 0.005^**^), Nowakowskiellaceae (-1.0, 0.025^*^), Stachybotryaceae (-1.0, 0.049^*^), *Nowakowskiella elegans* (-0.9, 0.028^*^)  Fungal taxon ^e^: Phymatotrichopsis (-2.0, 0.004^**^), Nowakowskiella (-1.0, 0.024^*^)  Fungal trophic mode ^e^: Pathotroph (-2.0, 0.006^**^), Pathotroph-saprotroph (1.1, 0.016^*^)  Fungal guilds ^e^: Plant pathogen (-1.5, 0.005^**^), Dung-plant saprotroph (-1.0, 0.050), Undefined saprotroph (-1.0, 0.040^*^) | |
| ———————————————————————————————————————————————————————— | |
| Burning effect: the relative abundance of the microbial group in the conventional tilled beds with residue incorporated (CBK, CBKN) versus that in the permanent beds with residue burned (PBB, PBBN) | |
| ———————————————————————————————————————————————————————— | |
| Fungi: *Aspergillus niger* (-1.4, 0.014^*^), Chytridiomycetes (0.9, 0.043^*^), uncultured *Ophiosphaerella* (1.1, 0.021^*^), Phaeosphaeriaceae (1.2, 0.023^*^), *Gelasinospora tetrasperma* (1.7, 0.004^**^)  Fungal taxon: Sordariaceae (-1.8, 0.006^**^), Phymatotrichopsis (-1.7, 0.004^**^), Ascodesmidaceae (-1.0, 0.038^*^)  Fungal guilds: Dung-plant saprotroph (-1.1, 0.016^*^), Dung-soil saprotroph (-1.0, 0.025^*^), Plant pathogen (-1.0, 0.044^*^) | |
| ——————————————————————————————————————————————————————— |  |

**Table S5** Continued.

———————————————————————————————————————————————————————

^a^ CBK: conventional tilled beds, maize-wheat rotation (MW) all crop residues incorporated by conventional tillage each crop cycle and new beds are formed after each crop (CB), CBKN: the same as CBK but with application of N fertilizer at 300 kg ha^-1^, PBB: permanent beds (PB beds are reshaped as needed for each crop) with wheat maize crop rotation, wheat and maize straw burned, PBBN: the same as PBB but with application of N fertilizer, PBK: permanent beds with straw retained, both wheat and maize straw chopped and left in place while permanent beds are reshaped as needed, PBKN: the same as PBK but with but with application of N fertilizer,  ^b^ The effect size, which is defined as the difference between groups divided by the maximum dispersion within group A or B, was calculated with the aldex.ttest argument (ALDEx2 (version, 1.18). A negative value indicates that the relative abundance of the microbial group was higher in the first considered treatments than in the second ones, ^c^ non-parametric Kruskal Wallis test was used to calculate the *p* value with the aldex.kw argument (ALDEx2 (version, 1.18), ^d^ *p* ≤ 0.05 and > 0.01, ^**^ ≤ 0.01 and > 0.001, ^e^ taxon, trophic mode and guild of the fungal groups were determined with the FUNGuildR package.

| ——————————————————————————————————————————————————————— |
| --- |
