# Supplementary material for: The Fungal and Protist Community as Affected by Tillage, Crop Residue Burning and N Fertilizer Application
Source: Curr Microbiol. 2025 Feb 19;82(4):144. doi: 10.1007/s00284-025-04112-5 (PMC11839885; doi:10.1007/s00284-025-04112-5)
Supplement: Supplementary file 8 — Supplementary file8 (DOCX 20 kb) [file 284_2025_4112_MOESM8_ESM.docx]

**Table S6** Comparison of the Hill numbers at *q* = 0, 1 and 2 based on the fungal and protist species. Effect of burning (permanent beds (PB) crop residue burned vs PB crop residue retained), N fertilizer (crop left unfertilized (0 kg urea-N ha^-1^) vs crop fertilized with 300 kg urea N ha^-1^) and tillage (PB with crop residue retained vs conventional tilled beds with crop residue incorporated) on Hill numbers was determined with a non-parametric analysis (Package WRS2 (v 1.1-6)).

| ⎯⎯⎯⎯⎯⎯⎯⎯⎯⎯⎯⎯⎯⎯⎯⎯⎯⎯⎯⎯⎯⎯⎯⎯⎯⎯⎯⎯⎯⎯⎯⎯⎯⎯⎯⎯⎯⎯ | | | | | | | |
| --- | --- | --- | --- | --- | --- | --- | --- |
| Microorganisms | Factor | Hill number | F value | *p* value | |  |  |
| ⎯⎯⎯⎯⎯⎯⎯⎯⎯⎯⎯⎯⎯⎯⎯⎯⎯⎯⎯⎯⎯⎯⎯⎯⎯⎯⎯⎯⎯⎯⎯⎯⎯⎯⎯⎯⎯⎯ | | | | | | | |
| Fungal species | Burning (Df ^a^ = 1, *n* = 6) | *q =* 0 | 0.17 | | 0.704 | |  |
|  |  | *q =* 1 | 0.03 | | 0.865 | |  |
|  |  | *q =* 2 | 0.17 | | 0.703 | |  |
|  | N fertilizer (Df = 1, *n* = 12) | *q =* 0 | 0.25 | | 0.628 | |  |
|  |  | *q =* 1 | 0.01 | | 0.936 | |  |
|  |  | *q =* 2 | 0.06 | | 0.808 | |  |
|  | Tillage (Df = 1, *n* = 6) | *q =* 0 | 0.26 | | 0.635 | |  |
|  |  | *q =* 1 | 0.24 | | 0.646 | |  |
|  |  | *q =* 2 | 0.39 | | 0.560 | |  |
| Protist species | Burning (Df = 1, *n* = 6) | *q =* 0 | 0.02 | | 0.907 | |  |
|  |  | *q =* 1 | 1.45 | | 0.276 | |  |
|  |  | *q =* 2 | 5.79 | | 0.059 | |  |
|  | N fertilizer (Df = 1, *n* = 12) | *q =* 0 | 0.41 | | 0.557 | |  |
|  |  | *q =* 1 | 0.45 | | 0.533 | |  |
|  |  | *q =* 2 | 1.27 | | 0.320 | |  |
|  | Tillage (Df = 1, *n* = 6) | *q =* 0 | 0.17 | | 0.704 | |  |
|  |  | *q =* 1 | 2.14 | | 0.200 | |  |
|  |  | *q* = 2 | 7.39 | | 0.047 | |  |
| ⎯⎯⎯⎯⎯⎯⎯⎯⎯⎯⎯⎯⎯⎯⎯⎯⎯⎯⎯⎯⎯⎯⎯⎯⎯⎯⎯⎯⎯⎯⎯⎯⎯⎯⎯⎯⎯⎯ | | | | | | | |

^a^ Df = degree of freedom.
