# Supplementary material for: The Fungal and Protist Community as Affected by Tillage, Crop Residue Burning and N Fertilizer Application
Source: Curr Microbiol. 2025 Feb 19;82(4):144. doi: 10.1007/s00284-025-04112-5 (PMC11839885; doi:10.1007/s00284-025-04112-5)
Supplement: Supplementary file 9 — Supplementary file8 (DOCX 22 kb) [file 284_2025_4112_MOESM9_ESM.docx]

Table S7 Effect of burning (permanent beds (PB) crop residue burned vs PB crop residue retained), N fertilizer (crop unfertilized (0 kg urea-N ha^-1^) vs crop fertilized with 300 kg urea N ha^-1^) and tillage (PB with crop residue retained vs conventional tilled beds with crop residue incorporated) on the microscopic eukaryotes, protists community, fungal and assigned guilds structure in soil at CIMMYT’s Norman E. Borlaug (CENEB) experimental station near Ciudad Obregon (Sonora, Mexico).

| ⎯⎯⎯⎯⎯⎯⎯⎯⎯⎯⎯⎯⎯⎯⎯⎯⎯⎯⎯⎯⎯⎯⎯⎯⎯⎯⎯⎯⎯⎯⎯⎯⎯⎯⎯⎯⎯ | | | | | | | | |
| --- | --- | --- | --- | --- | --- | --- | --- | --- |
|  | Roots | | | | | | |  |
|  | ⎯⎯⎯⎯⎯⎯⎯⎯⎯⎯⎯⎯⎯⎯⎯⎯⎯⎯⎯⎯⎯⎯ | | | | | | |  |
|  | Seminal ^a^ | | Crown ^b^ | | | Tiller ^c^ | |  |
|  | ⎯⎯⎯⎯⎯⎯⎯ | | ⎯⎯⎯⎯⎯⎯⎯ | | | ⎯⎯⎯⎯⎯⎯⎯ | |  |
| Comparison | F value ^d^ | *p* value | F value | | *p* value | F value | *p* value |  |
| ⎯⎯⎯⎯⎯⎯⎯⎯⎯⎯⎯⎯⎯⎯⎯⎯⎯⎯⎯⎯⎯⎯⎯⎯⎯⎯⎯⎯⎯⎯⎯⎯⎯⎯⎯⎯⎯ | | | | | | | | |
| All years |  |  |  | |  |  |  |  |
| N fertilizer (Df ^e^ = 1, *n* = 6) | 11.18 | 0.002^** f^ | | 21.21 | 0.001^***^ | 21.59 | 0.001^***^ |  |
| Tillage (Df = 1, *n* = 6) | 1.93 | 0.172 | 3.83 | | 0.057 | 3.65 | 0.063 |  |
| Burning (Df = 1, *n* = 6) | 1.04 | 0.749 | 0.92 | | 0.342 | 3.78 | 0.059 |  |
| 2012 |  |  |  | |  |  |  |  |
| N fertilizer (Df = 1, *n* = 6) | 123.32 | 0.001^***^ | 20.47 | | 0.003^**^ | 12.26 | 0.008^**^ |  |
| Tillage (Df = 1, *n* = 6) | 0.68 | 0.442 | 2.21 | | 0.190 | 0.69 | 0.462 |  |
| Burning (Df = 1, *n* = 6) | 0.00 | 1.000 | 0.00 | | 1.000 | 1.35 | 0.298 |  |
| ⎯⎯⎯⎯⎯⎯⎯⎯⎯⎯⎯⎯⎯⎯⎯⎯⎯⎯⎯⎯⎯⎯⎯⎯⎯⎯⎯⎯⎯⎯⎯⎯⎯⎯⎯⎯⎯ | | | | | | | | |

^a^ Any of the adventitious roots (plant roots that form from any non-root tissue) that grow from the base of the stem during early seedling growth and take over the functions of the radicle (<http://www.botanydictionary.org/seminal-root.html>), ^b^ root crown: part of a root system from which a stem arises (<https://en.wikipedia.org/wiki/Root_crown>), ^c^ tiller root: roots of a shoot that arises from the base of a grass plant (<https://en.wikipedia.org/wiki/Tiller_(botany)>, ^d^ Non-parametric analysis with the WRS2 Package (v 1.1-0), ^e^ Df = degree of freedom, ^f^ ^*^ *p* ≤ 0.05 and > 0.01, ^**^ *p* ≤ 0.01 and > 0.001, ^***^ *p* ≤ 0.001.

| ⎯⎯⎯⎯⎯⎯⎯⎯⎯⎯⎯⎯⎯⎯⎯⎯⎯⎯⎯⎯⎯⎯⎯⎯⎯⎯⎯⎯⎯⎯⎯⎯⎯⎯⎯⎯⎯ |
| --- |
